# Effect of Transverse Aortic Constriction on Cardiac Structure, Function and Gene Expression in Pregnant Rats

**DOI:** 10.1371/journal.pone.0089559

**Published:** 2014-02-20

**Authors:** Nils Thomas Songstad, David Johansen, Ole-Jacob How, Per Ivar Kaaresen, Kirsti Ytrehus, Ganesh Acharya

**Affiliations:** 1 Women's Health and Perinatology Research Group, Department of Clinical Medicine, Faculty of Health Sciences, University of Tromsø, Tromsø, Norway; 2 Department of Pediatrics, University Hospital of Northern Norway, Tromsø, Norway; 3 Cardiovascular Research Group, Department of Medical Biology, Faculty of Health Sciences, University of Tromsø, Tromsø, Norway; 4 Pediatric Research Group, Department of Clinical Medicine, Faculty of Health Sciences, University of Tromsø, Tromsø, Norway; 5 Department of Obstetrics and Gynecology, University Hospital of Northern Norway, Tromsø, Norway; University of Otago, New Zealand

## Abstract

**Background:**

There is an increased risk of heart failure and pulmonary edema in pregnancies complicated by hypertensive disorders. However, in a previous study we found that pregnancy protects against fibrosis and preserves angiogenesis in a rat model of angiotensin II induced cardiac hypertrophy. In this study we test the hypothesis that pregnancy protects against negative effects of increased afterload.

**Methods:**

Pregnant (gestational day 5.5–8.5) and non-pregnant Wistar rats were randomized to transverse aortic constriction (TAC) or sham surgery. After 14.2±0.14 days echocardiography was performed. Aortic blood pressure and left ventricular (LV) pressure-volume loops were obtained using a conductance catheter. LV collagen content and cardiomyocyte circumference were measured. Myocardial gene expression was assessed by real-time polymerase chain reaction.

**Results:**

Heart weight was increased by TAC (p<0.001) but not by pregnancy. Cardiac myocyte circumference was larger in pregnant compared to non-pregnant rats independent of TAC (p = 0.01), however TAC *per se* did not affect this parameter. Collagen content in LV myocardium was not affected by pregnancy or TAC. TAC increased stroke work more in pregnant rats (34.1±2.4 vs 17.5±2.4 mmHg/mL, p<0.001) than in non-pregnant (28.2±1.7 vs 20.9±1.5 mmHg/mL, p = 0.06). However, it did not lead to overt heart failure in any group. In pregnant rats, α-MHC gene expression was reduced by TAC. Increased in the expression of β-MHC gene was higher in pregnant (5-fold) compared to non-pregnant rats (2-fold) after TAC (p = 0.001). Nine out of the 19 genes related to cardiac remodeling were affected by pregnancy independent of TAC.

**Conclusions:**

This study did not support the hypothesis that pregnancy is cardioprotective against the negative effects of increased afterload. Some differences in cardiac structure, function and gene expression between pregnant and non-pregnant rats following TAC indicated that afterload increase is less tolerated in pregnancy.

## Introduction

Pregnancy leads to profound physiological changes in the cardio-vascular system that are generally reversible. Pregnancy induced changes in estrogens, progesterones [1], [2], relaxin [3] and mineralocorticoids [4] mainly contribute to this process.

Failure of cardiovascular system to adapt to physiological changes of pregnancy may lead to hypertensive disorders, which are frequently associated with adverse outcomes [5]. Furthermore, advances in cardiac surgery and improvement in the care of children with congenital heart defects has led to a growing number of pregnant women with Grown-Up Congenital Heart (GUCH) defects including conditions associated with increased afterload, such as aortic stenosis and coarctation of the aorta [6], [7]. Knowledge of how pregnancy influences cardiac structure and function is crucial for providing optimal care for pregnant women with hypertension or cardiac disease.

In a previous study we found that the pregnancy protects against fibrosis and preserves angiogenesis in a rat model of angiotensin II (AngII) induced cardiac hypertrophy. Pregnancy also led to modulation of the gene expression profile of the AngII exposed hearts [8]. However, contrary to what is seen in mice [9] and humans [10], pregnancy *per se* did not lead to significant hypertrophy in the rat hearts [8]. Few studies have looked at how gene expression in the heart is affected by pregnancy [1]. Studies in mice have shown that the molecular signature of heart hypertrophy in pregnancy is different from that of pathological hypertrophy [9]. However, experimental studies on how pregnancy affects the heart's structural and functional adaptation to increased afterload are lacking.

The aim of this study was to investigate the effect of isolated chronic pressure load on the hearts of pregnant rats. Based on our previous finding that pregnancy may protect the heart against negative effects of chronic AngII infusion [8], we decided to test the hypothesis that pregnancy is protective against the negative effects of increased cardiac afterload.

## Materials and Methods

### Ethics Statement

Animal experiments conformed to the Directive 2010/63/EU of the European Parliament on the protection of animals used for scientific purposes [11] and all procedures were approved by the Norwegian Committee on Ethics in Animal Experimentation (project ID 2177).

### Animals and model

Female Wistar rats with mean body mass 208±22 grams of similar age (Charles River, Sulzfeld, Germany) were used in this study. The animals were housed in cages in pairs under controlled conditions of temperature, light-dark periods of 12 h, and free access to water and standard diet. Pregnant rats were obtained by mating with a male rat housed together with two females overnight in a cage Mating was confirmed next morning by the presence of a vaginal plug, and the day of the vaginal plug was considered gestational day (GD) 0.5.

On GD5.5–8.5 (mean 6.4) the animals were randomized to either transverse aorta constriction (TAC) or sham surgery. Anesthesia was induced with isoflurane 4% in an induction chamber. The rats were then intubated and ventilated (New England Medical Instruments Inc., Medway, MA, USA) delivering tidal volumes of 2–3 ml at a frequency of 60 per minute. 2.5% isoflurane in 100% oxygen (Vevo Compact Anesthesia System, VisualSonics, Toronto, Canada) was used to maintain the anesthesia. The rats were placed supine on a warm electric pad and the temperature was kept stable at approximately 38°C. The heart rate and the rectal temperature were monitored continuously. A heating lamp was used when required. Buprenorphine (Temgesic, Reckitt Benckiser, UK) 0.05 mg/kg was administered subcutaneously and bupivacaine (Marcain, AstraZeneca, Sweden) locally for analgesia. Hair was removed with a mechanical shaver and application of depilatory cream. Pain-reflexes were checked before surgery was started and isoflurane increased if necessary. After skin incision, the upper half of the sternum was divided in the midline using scissors and thymus was removed. The aortic arch was carefully dissected free of the surrounding tissues. A bended and blunted stylet from a 16 or 18G intravenous (IV) catheter (Optiva, Smith Medical International Ltd., Rossendale, UK) was tied tightly to the aorta between the brachiocephalic trunk and the left common carotid artery using 4–0 silk (SofSilk, Synture, Mansfield, MA, USA) and then removed, creating partial aortic constriction. The sternotomy and the skin incision were closed with 5–0 sutures (Polysorb, Synture, Mansfield, MA, USA). The rats were extubated and put in an incubator (Vetario S10 Intensive Care Unit, Brinsea Products Ltd, N. Somerset, UK) at 28–30°C for the recovery period. Sham operated animals underwent exactly the same procedure except ligation of the aorta. Postoperatively the animals were kept in separate cages and allowed free access to water and standard laboratory diet. Analgesia with buprenorphine 0.05 mg/kg subcutaneously was provided every 12 hour for 48 hours.

Acute experiments were performed 14 days (range, 13–17) after TAC. In pregnant rats this corresponded to GD20.5±0.3 (term  = 21–22 days in rats). Anesthesia was provided with inhaled isoflurane 1.5% in 100% oxygen with temperature control and monitoring as described earlier.

### Transthoracic echocardiography

Echocardiography was performed using a high resolution ultrasound imaging system equipped with a RMV-710B transducer with a frequency of 25 MHz and a fixed focal length of 15 mm mounted on an integrated rail system (Vevo 770, Visualsonics, Toronto, Canada). Prewarmed ultrasound gel was used. M-mode recordings were obtained from the parasternal short-axis views. All ultrasound based measurements were performed off-line without the knowledge of the animals' identity. Care was taken to select three consecutive cycles with good quality signals. The internal dimensions of the LV cavity and thickness of the anterior and posterior LV walls were measured. The heart rate (HR) was obtained from the electro-cardiogram signals. LV fractional shortening % was calculated as 100× ((LVIDd-LVIDs)/LVIDd) where, LVIDd  =  LV internal diameter in diastole and LVIDs  =  LV internal diameter in systole. Stroke volume (SV) was calculated as LV EDV – ESV where, EDV  =  end diastolic volume and ESV  =  end systolic volume. The LV EDV was calculated as 7.0/(2.4+ LVIDd)xLVIDd^3^ and the LV ESV was calculated as 7.0/(2.4+ LVIDs)xLVIDs^3^ [12], and cardiac output per minute (CO) was calculated as SV × HR. Relative wall thickness (RWT) was calculated using the formula: RWT  =  (LVPWd + LVAWd)/LVIDd where, LVPWd =  LV posterior wall thickness and LVAWd  =  LV anterior wall thickness. LV mass was calculated using the formula: LV mass  = 1.04×(LVIDd + LVPWd + LVAWd)^3^– LVIDd^3^ [13].

### Invasive measurements of blood pressure and LV volume

Following echocardiography a 2F microtip pressure-volume (PV) catheter (SPR-838; Millar Instruments Inc, Houston, TX, USA) was inserted into the ascending aorta via the right carotid artery. Aortic blood pressures (BP) were measured before the catheter was introduced into the LV. The PV-signal was recorded by a PowerLab using a LabChart 7 acquisition system (AD Instruments) and was used to verify proper position of the catheter in the LV. The animal was allowed to stabilize before the baseline PV-loops were recorded. PVAN 3.6 software (Millar Instruments Inc) was used to analyze PV-loop data. Raw signals from volume measurements were calibrated with SV calculated from M-mode echocardiography. Mean arterial pressure (MAP) was calculated as 2/3× diastolic BP +1/3× systolic BP, total peripheral resistance (TPR) as MAP (minus end-diastolic pressure) divided by CO, and LV stroke work was calculated as the difference between maximum and minimum LV pressure multiplied with SV. Effective arterial elastance (Ea) was calculated as the ratio of LV end-systolic pressure to SV. dP/dt_max_, dP/dt_min_ and isovolumetric relaxation constant (Tau) were calculated by the software. LV end-systolic elastance (Ees) was calculated as the ratio of end systolic pressure to ESV.

In the end of experiment, the animals were euthanized with sodium pentobarbital 100 mg/kg administered intravenously or intraperitoneally, and the hearts were excised immediately. The heart, LV, fetuses and placentas were weighed and tibia length and fetal crown-rump length were measured. Tissue samples were collected from LV and were used for the analysis of myocardial gene expression, collagen content and cardiomyocyte size. All analyses were performed in a blinded fashion without the knowledge of TAC and pregnancy status.

### Measurement of myocyte circumference and collagen content

**Figure 1:**
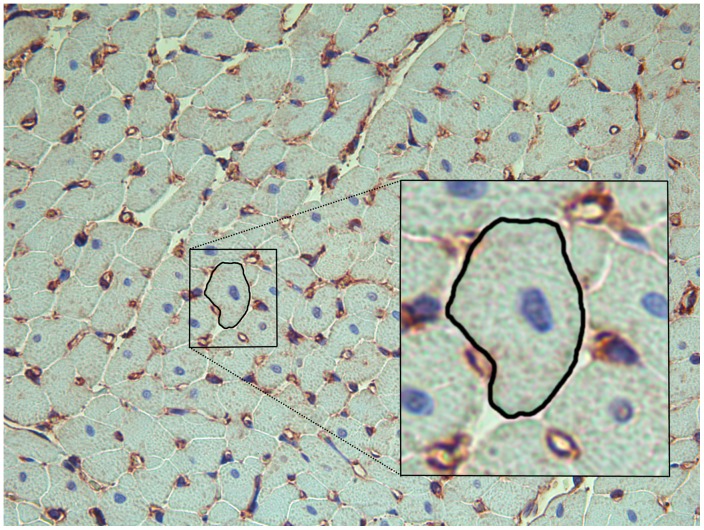
Light microscopy of heart tissue. Histological section of heart tissue (200x) stained with toluidine blue to enhance the contour of the cardiomyocytes for measuring their circumference and immunohistochemistry using non-muscular β-actin to stain capillaries (brown). One cardiomyocyte cut in short axis and containing a nucleus (blue) is outlined and magnified (box) to show how cardiomyocyte circumference was measured (in arbitrary units).

Myocardial tissue samples were taken from the septum and free wall of the middle part of the LV and fixed in formalin. Slides stained with toluidine blue were used for the measurement of cardiomyocyte circumference (Fig. 1), and with Sirius Red for the measurement of collagen content, as previously described [8]. A Leica DM2000 microscope was used for viewing slides, and photographs were taken with a Leica DFC 425 digital camera (Leica Microsystems, Wetzlar, Germany). Sixteen photos from each slide were acquired. Cardiomyocytes cut in short axis and containing a nucleus (n = 97±7) were identified from each heart and cell circumference was measured. The cell was outlined digitally using Image J (National Institutes of Health, Bethesda, MD, USA) and circumference was expressed in arbitrary units. Image J was used to analyze % tissue area stained by Sirius Red as described online by the developer [14].

### Gene expressions

Apical myocardial tissue was sampled and stored for gene expression analyses using quantitative real-time polymerase chain reaction as described previously [8]. Nineteen genes with potential relation to cardiac remodeling were selected: Atrial natriuretic peptide (ANP), brain natriuretic peptide (BNP), ankyrin repeat domain-containing protein 1 (ANKRD1), α-myosin heavy chain (α-MHC), β-MHC, protein kinase Cα (PKCα), tumor necrosis factor α (TNFα), collagen type I-a 1(COL1A1), collagen type III-a 1 (COL3A1), fibronectin (FN1), tissue inhibitor of metallopeptidase 1(TIMP1), vascular endothelial growth factor A and B (VEGF-A and -B), transforming growth factor β1 (TGFβ1), TGFβ2, TGFβ3, superoxide dismutase 1 (SOD1), inducible nitric oxide synthase (iNOS) and endothelial NOS (eNOS). The primers are given in Table S1.

### Statistics

All data are reported as mean±standard error of the mean (SEM). *P*<0.05 was considered statistically significant. Logarithmic transformation was used to achieve normal distribution of continuous variables when appropriate. Correlation between parametric variables was checked using Pearson Correlation coefficient. One way analysis of variance (ANOVA) was used to compare groups and two way ANOVA was used to investigate the influence and interaction between pregnancy status and TAC. Only differences in influence without significant interaction between pregnancy and TAC are presented for two way ANOVA. The Holm–Sidak method was used as post-hoc test. PASW Statistics 18.0.3 (SPSS Inc., Chigaco, IL, USA) and Sigma Plot 12.0 (Systat Software Inc, San Jose, CA, USA) softwares were used for statistical analyses.

## Results

A total of 57 female rats were used for this study. Seven animals died during surgery. Three rats that had TAC using a stylet from an 18G IV catheter (tighter aorta constriction) developed acute heart failure (dyspnoea, cyanosis and hemoptysis) postoperatively and were euthanized. Two rats delivered before the experiments were performed and three were excluded due to inappropriate banding time. Thus a total of 42 animals were included in the final analysis. Fifteen of these underwent TAC using stylet from a 16G IV catheter and four rats (two pregnant and two non-pregnant) using stylet from an 18G IV catheter. The weights of the animals included are presented in Table 1. There was no statistically significant difference in weight gain after surgery in TAC animals compared to sham.

**Table 1:** Biometric data and parameters of cardio-vascular function measured using echocardiography and conductance catheter.

	Non-pregnant sham (n)	Non-pregnant TAC (n)	Pregnant sham (n)	Pregnant TAC (n)
Body weight (g)	220±7 (11)	226±6 (11)	274±6^*†^ (11)	272±10^*†^ (9)
Left ventricular weight (mg)	423±15 (9)	538±37^*^ (10)	389±13^†^ (9)	572±39^*‡^ (7)
Heart rate (/min)	408±12 (10)	425±10 (10)	426±9 (11)	421±10 (8)
Fractional shortening (%)	45±3 (9)	49±3 (9)	49±1 (10)	47±3 (9)
Cardiac output (mL/min)	64±3 (8)	68±3 (8)	69±4 (10)	65±5 (8)
Left ventricular inner diameter in diastole (mm)	6.4±0.1 (9)	6.3±0.1 (9)	6.3±0.1 (10)	6.3±0.2 (9)
Left ventricular relative wall thickness (%)	43±3 (9)	54±3 (9)	49±1 (10)	56±6 (9)
Calculated left ventricular mass (mg)	544±25 (9)	704±53 (9)	625±25 (10)	714±57^*^ (9)
Left ventricular posterior wall thickness in diastole (mm)	1.51±0.10 (9)	1.82±0.11 (9)	1.62±0.06 (10)	1.78±0.20 (9)
Sum of septum and posterior wall thickness in diastole (mm)	2.77±0.12 (9)	3.40±0.16 (9)	3.11±0.06 (10)	3.44±0.24^*^ (9)
Total peripheral resistance (mmHg*min/mL)	1.68±0.13 (8)	1.92±0.18 (8)	1.20±0.08^†^ (10)	1.83±0.12^‡^ (6)
dP/dt_max_ (mmHg/sec*10^3^)	10.9±0.7 (11)	10.8±0.6 (8)	7.7±1.1^*^ (6)	11.0±0.7 (5)
dP/dt_min_ (mmHg/sec*10^3^)	−12.2±0.9 (11)	−13.2±0.6 (8)	−8.7±1.3^†^ (6)	−15.9±1.3^‡^ (5)
Isovolumetric relaxation constant (msec)	10.6±1.0 (11)	10.2±0.6 (9)	10.1±0.6 (6)	8.8±0.2 (5)
Arterial elastance (mmHg/mL)	0.83±0.06 (9)	0.98±0.09 (7)	0.58±0.02^†^ (5)	1.24±0.11^*‡^ (5)
End-systolic elastance (mmHg/mL)	2.05±0.25 (9)	4.37±1.3 (7)	2.31±0.30 (5)	5.23±1.5 (5)

Data are presented as mean±SEM. Comparisons between groups were made using one-way ANOVA and Holm-Sidak post hoc test. TAC, transverse aorta constriction. n, number of animals. p<0.05 compared to non-pregnant sham (*), non-pregnant TAC (†), or pregnant sham rats (‡).

### TAC increases proximal aortic blood pressure and leads to cardiac hypertrophy in pregnant rats

**Figure 2:**
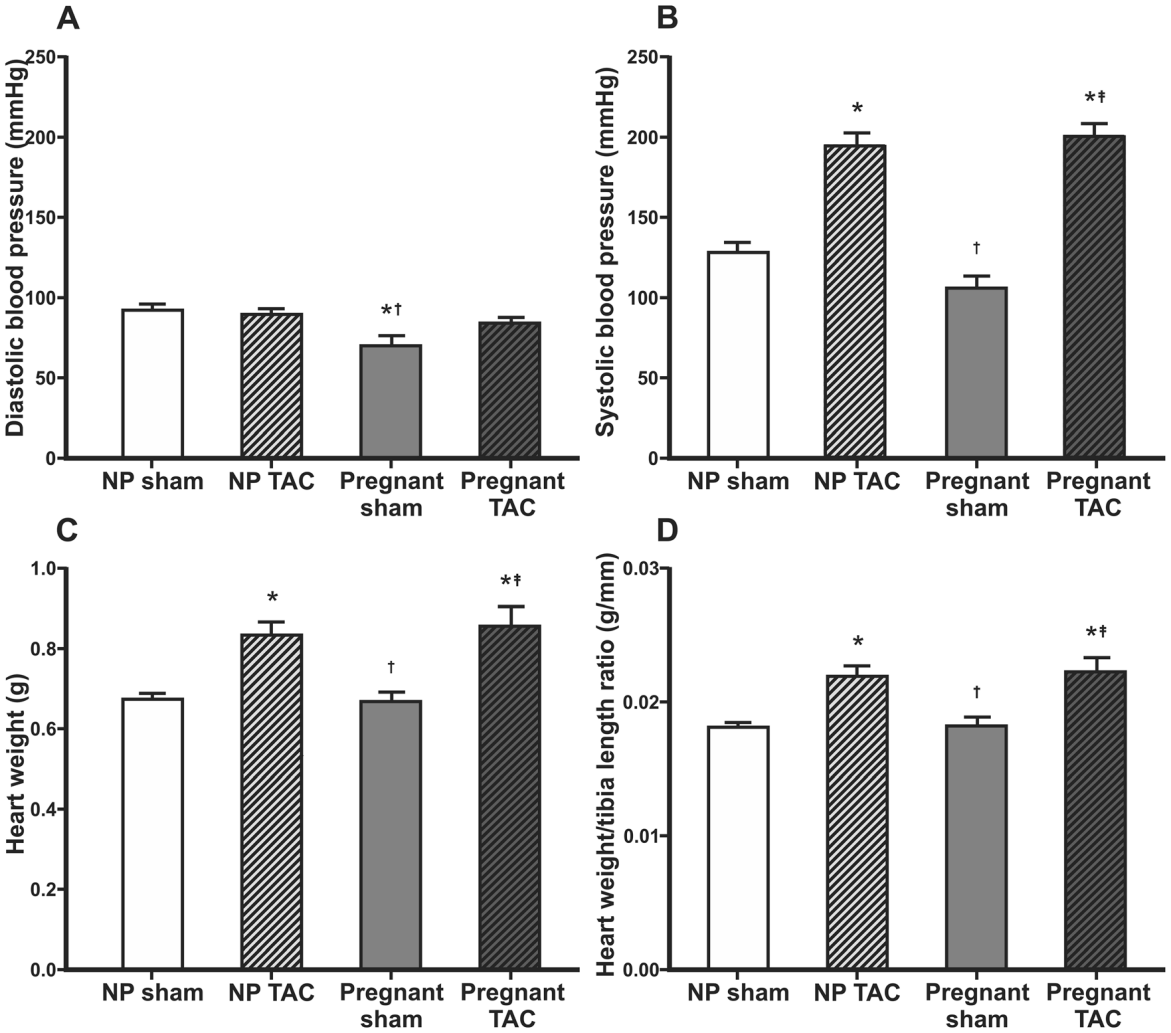
Blood pressure, heart weight and heart weight/tibia length ratio. Mean±SEM for diastolic (A) and systolic (B) blood pressure in ascending aorta, heart weight (C) and heart weight/tibia length ratio (D). Comparisons between groups were made using one-way ANOVA and Holm-Sidak post hoc test. NP, non-pregnant, TAC, transverse aorta constriction. p<0.05 compared to non-pregnant sham (*), non-pregnant TAC (†), or pregnant sham rats (‡).

Systolic BP proximal to the banding site was markedly increased in both TAC groups compared to sham groups, whereas diastolic BP was not affected by TAC. BP was decreased in pregnant compared to non-pregnant sham operated rats (Fig. 2).

**Figure 3:**
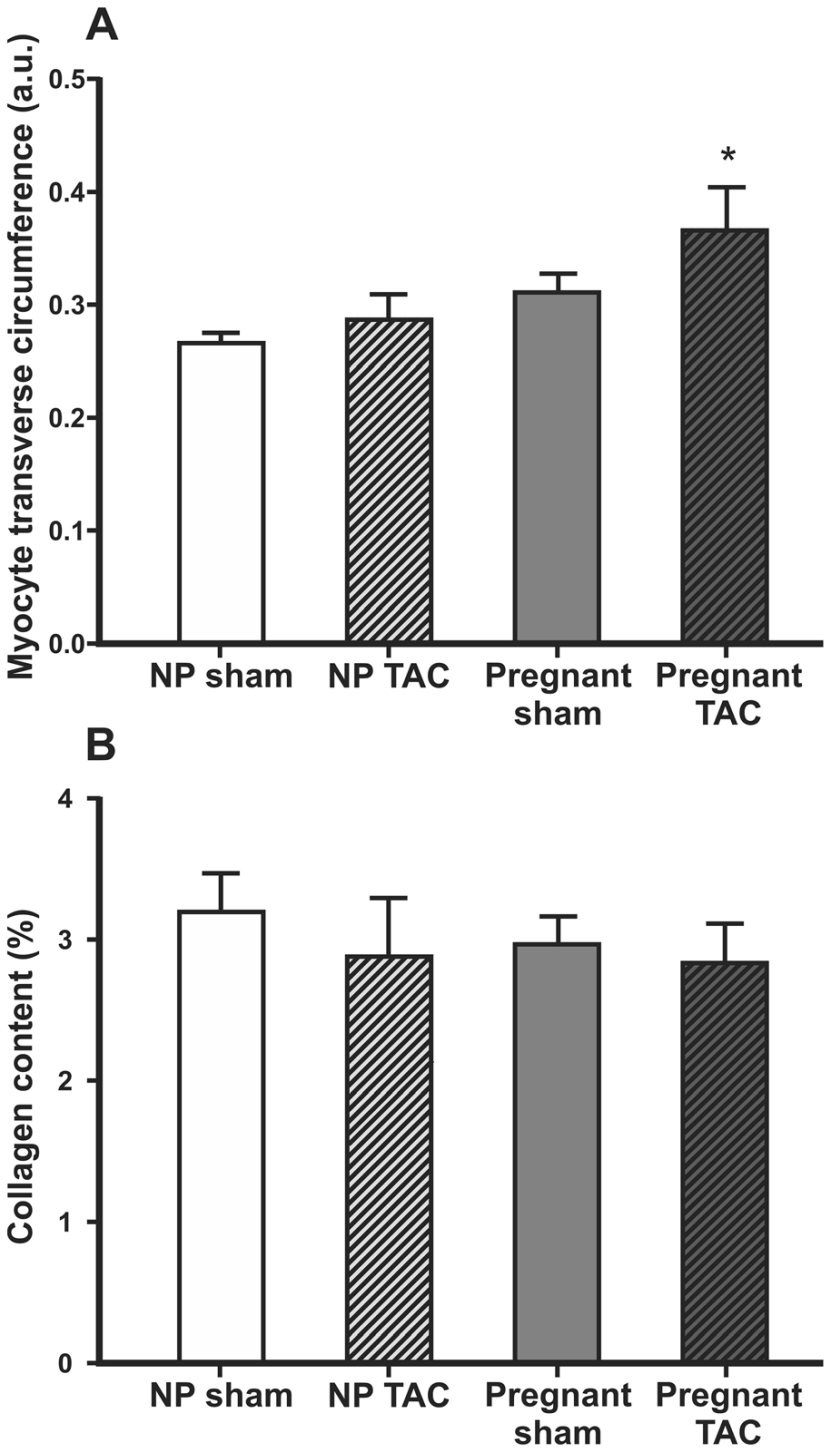
Myocyte transverse circumference and collagen content in myocardial tissue. Myocyte transverse circumference (A) expressed in arbitrary units (a.u.) and collagen content in myocardial tissue (B) expressed as % tissue area stained by Sirius Red presented as mean±SEM. Comparisons between groups were made using one-way ANOVA and Holm-Sidak post hoc test. NP, non-pregnant, TAC, transverse aorta constriction. p<0.05 compared to non-pregnant sham (*).

Heart weight (HW), LV weight, and HW/tibia ratio did not differ between pregnant and non-pregnant sham animals, but were increased in both TAC groups (Fig. 2 and Table 1). Heart weight/body weight (HW/BW) ratio was higher in TAC compared to sham and lower in pregnant rats compared to sham. TAC did not affect body weight gain postoperatively in pregnant or non-pregnant rats. Circumference of myocyte transverse sections and collagen content within the myocardium are presented in Fig. 3. Myocyte transverse circumference correlated with HW (r = 0.60, p = 0.001), LV weight (r = 0.66, p<0.001) and HW/tibia-ratio (r = 0.51, p = 0.005). However, when myocyte circumference was compared between groups, significant difference was found only between the pregnant TAC and non-pregnant sham animals (Fig. 3). Cardiomyocytes of the pregnant rats had a significantly larger circumference than that of the non-pregnant rats when analysis was performed independent of TAC (p = 0.01). Myocardial tissue collagen content was not affected by pregnancy or TAC (Fig. 3).

### TAC increased contractile function and did not lead to heart failure

**Figure 4:**
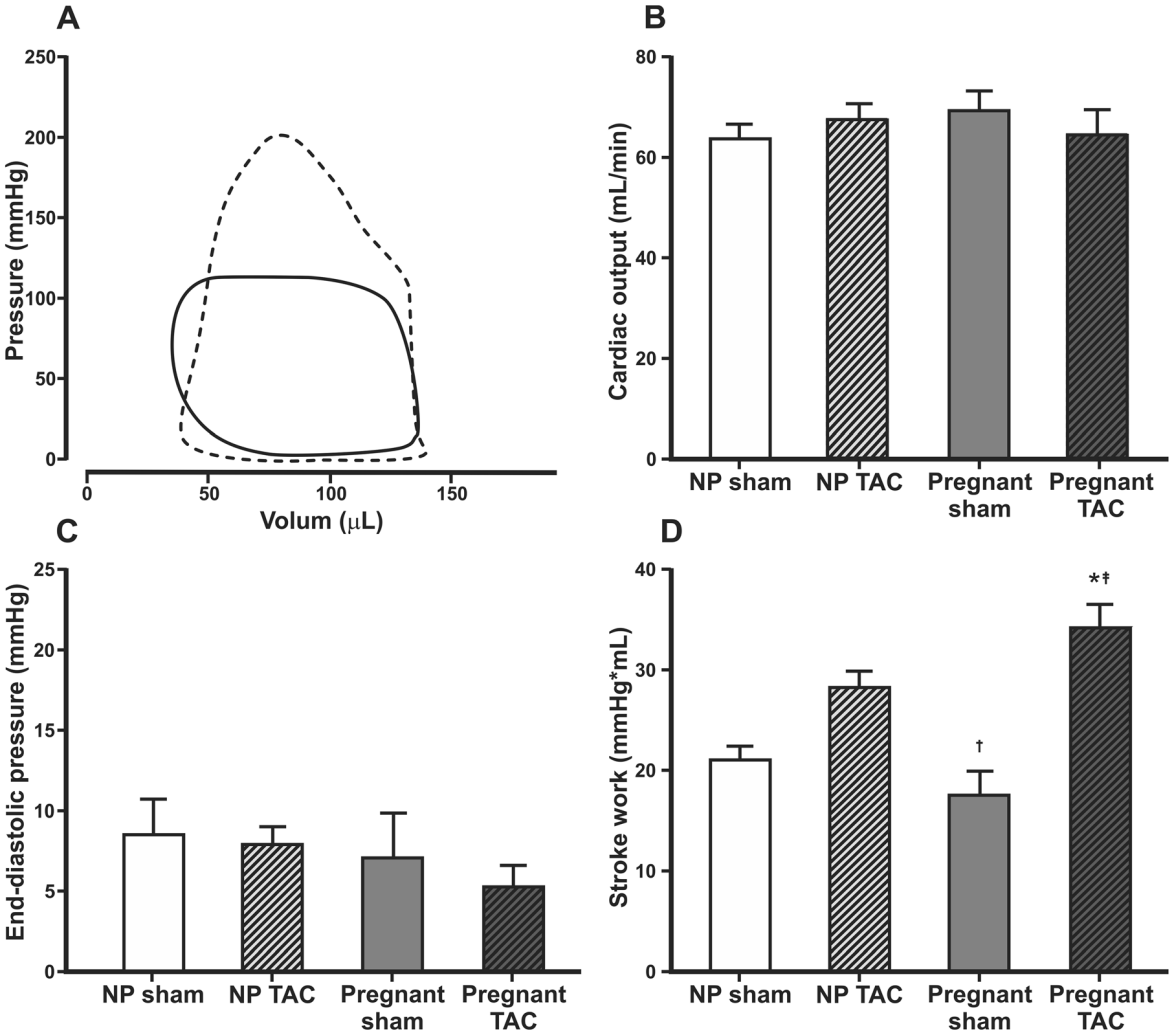
Pressure-volume loops, cardiac output, end-diastolic pressure and stroke work. Typical pressure-volume loops obtained by conductance catheter from a sham operated non-pregnant rat (full line) and a pregnant rat with transverse aortic constriction (dotted line) (A). Mean±SEM for calculated cardiac output (B), end-diastolic pressure (C) and calculated stroke work (D). Comparisons between groups were made using one-way ANOVA and Holm-Sidak post hoc test. NP, non-pregnant, TAC, transverse aorta constriction. p<0.05 compared to non-pregnant sham (*), non-pregnant TAC (†), or pregnant sham rats (‡).

M-mode echocardiographic parameters of cardiac function were not significantly different between groups (Table 1). Typical PV-loops, cardiac output, end-diastolic pressure and calculated stroke work are presented in Fig. 4. There were no differences in filling pressures between groups, indicating that TAC for ∼2 weeks did not lead to heart failure. TAC increased stroke work in pregnant rats (34.1±2.4 vs 17.5±2.4 mmHg/mL, p<0.001) but this was not significant in non-pregnant animals (28.2±1.7 vs 20.9±1.5 mmHg/mL, p = 0.06).

### Pregnancy affects genes associated with cardiac remodeling

Differences in gene expression related to cardiac remodeling among different study groups are presented in Table 2, and how their expression is influenced by pregnancy and TAC is shown in Table 3. α-MHC gene expression was decreased by TAC (p = 0.006) and pregnancy (p<0.001) independent of each other, whereas expression of β-MHC was higher in pregnant TAC than in non-pregnant TAC (p = 0.001). The ratio of β-MHC to α-MHC expression was higher in pregnant TAC compared to non-pregnant TAC (7.5±1.2 vs. 2.2±0.4, p<0.001). ANP and BNP were increased by TAC (p<0.001) but not affected by pregnancy. TNF-α was increased (p = 0.03) by TAC in non-pregnant rats only and reduced (p<0.001) by pregnancy in TAC only. Expression of the angiogenesis related genes VEGF-α and VEGF-β were not affected by TAC but reduced by pregnancy. Of the genes related to oxidative stress, SOD1 was not affected and eNOS was decreased by pregnancy (p = 0.002). iNOS was increased by TAC (p = 0.002) in non-pregnant rats and decreased by pregnancy in TAC (p<0.001). Expression of fibrosis related genes (COL1A1, COL3A1, FN1 and TIMP1) was up-regulated by TAC. Pregnancy increased the expression of TIMP1 independent of TAC (p = 0.02).

**Table 2:** Myocardial gene expression.

	Non-pregnant sham (n = 7)	Non-pregnant TAC (n = 7)	Pregnant sham (n = 7)	Pregnant TAC (n = 7)
**Heart function related genes**				
PKCα	1.00±0.04	1.21±0.08	0.84±0.04^†^	1.03±0.03^†^
αMHC	1.00±0.05	0.94±0.05	0.88±0.04	0.68±0.04^*†‡^
βMHC	1.00±0.15	2.09±0.48	0.95±0.11	5.04±0.78^*†‡^
ANP	1.00±0.08	3.63±0.99	1.18±0.26	4.74±0.91*^†^
BNP	1.00±0.20	2.46±0.59	0.58±0.09	2.53±0.73^‡^
ANKRD1	1.00±0.18	1.46±0.20	0.54±0.02^†^	1.18±0.15^‡^
TNFα	1.00±0.09	1.41±0.07^*^	0.85±0.12^†^	0.76±0.08^†^
TGFβ1	1.00±0.04	1.32±0.06^*^	0.85±0.05^†^	1.19±0.08^‡^
TGFβ2	1.00±0.17	1.54±0.25	0.74±0.05^†^	1.45±0.24
TGFβ3	1.00±0.03	1.19±0.10	0.83±0.03^†^	0.91±0.04^†^
**Angiogenesis related genes**				
VEGFα	1.00±0.05	1.00±0.07	0.76±0.03^*†^	0.91±0.07
VEGFβ	1.00±0.07	1.11±0.06	0.88±0.04	0.87±0.06
**Oxidative stress related genes**				
SOD1	1.00±0.07	0.92±0.04	0.82±0.06	0.90±0.04
eNOS	1.00±0.05	1.21±0.09	0.88±0.07^†^	0.78±0.09^†^
iNOS	1.00±0.06	1.77±0.19^*^	0.54±0.10^†^	0.76±0.13^†^
**Fibrosis related genes**				
COL3A1	1.00±0.07	1.78±0.16^*^	0.93±0.11^†^	1.33±0.14*^‡^
COL1A1	1.00±0.10	1.83±0.15^*^	1.02±0.16^†^	1.56±0.21
FN1	1.00±0.04	1.18±0.09	0.98±0.10	1.52±0.16*^‡^
TIMP1	1.00±0.08	1.69±0.22	1.37±0.19	2.94±0.62*^‡^

Relative expression of genes related to cardiac remodeling normalized to mean values in non-pregnant sham operated animals. TAC, transverse aorta constriction, PKCα, protein kinase Cα, MHC, myosin heavy chain, ANP, atrial natriuretic peptide, BNP, brain natriuretic peptide, ANKRD1, ankyrin repeat domain-containing protein 1, TNFα, tumor necrosis factor α, TGF, transforming growth factor, VEGF, vascular endothelial growth factor, SOD1, superoxide dismutase 1, eNOS, endothelial nitric oxide synthase, iNOS, inducible nitric oxide synthase, COL1A1, collagen type I-a 1, COL3A1, collagen type III-a 1, FN1, fibronectin and TIMP1, tissue inhibitor of metallopeptidase 1. Data are presented as mean±SEM. Comparisons between groups were made using one-way ANOVA and Holm-Sidak post hoc test. p<0.05 compared to non-pregnant sham (*), non-pregnant TAC (†), or pregnant sham rats (‡).

**Table 3:** Influence of pregnancy and TAC on myocardial gene-expression.

	Influence of pregnancy independent of TAC	Influence of TAC independent of pregnancy
**Heart function related genes**		
PKCα	↓	↑↑
αMHC	↓↓	↓
βMHC	na	na
ANP	0	↑↑
BNP	0	↑↑
ANKRD1	↓	↑↑
TNFα	na	na
TGFβ1	↓	↑↑
TGFβ2	0	↑↑
TGFβ3	↓↓	↑
**Angiogenesis related genes**		
VEGF-α	↓	0
VEGF-β	↓	0
**Oxidative stress related genes**		
SOD1	0	0
eNOS	↓	0
iNOS	na	na
**Fibrosis related genes**		
COL3A1	0	↑↑
COL1A1	0	↑↑
FN 1	0	↑
TIMP1	↑	↑↑

Influence of pregnancy and transverse aorta constriction (TAC) on the expression of genes related to cardiac remodeling in apical myocardium of rats. Data were analyzed using two-way ANOVA and Holm-Sidak post hoc test. PKCα, protein kinase Cα, MHC, myosin heavy chain, ANP, atrial natriuretic peptide, BNP, brain natriuretic peptide, ANKRD1, ankyrin repeat domain-containing protein 1, TNFα, tumor necrosis factor α, TGF, transforming growth factor, VEGF, vascular endothelial growth factor, SOD1, superoxide dismutase 1, eNOS, endothelial nitric oxide synthase, iNOS, inducible nitric oxide synthase, COL1A1, collagen type I-a 1, COL3A1, collagen type III-a 1, FN1, fibronectin and TIMP1, tissue inhibitor of metallopeptidase 1. 0, no significant difference, ↑ increase p<0.05, ↑↑ increase p<0.001, ↓ decrease p<0.05, ↓↓ decrease p<0.001, na, non-applicable due to interaction.

### Fetuses were not affected by TAC

Out of eighteen pregnancies examined on GD20.5, there were no significant differences in size of litters, fetal weight, placental weight, crown-rump length or placenta/fetal weight ratio between sham operated and TAC animals (Table 4).

**Table 4:** Fetal outcome.

	Pregnant sham (n = 10)	Pregnant TAC (n = 8)	p-value
Number of fetuses	6.8±1.0	7.5±1.4	0.7
Fetal weight (g)	4.3±0.2	4.7±0.3	0.3
CRL (mm)	39±1	40±1	0.3
Placenta weight (g)	0.53±0.02	0.60±0.08	0.4
Placenta/fetal weight-ratio	0.124±0.006	0.127±0.013	0.9

Number of fetuses per animal, mean fetal weight per animal, mean crown-rump-length (CRL) per animal, mean placenta weight per animal and placenta weight/fetal weight-ratio per animal presented as mean±SEM.

## Discussion

We used an experimental model of TAC in rats to study the effects of chronic pressure load on cardiac remodeling in pregnancy. To our knowledge no such studies have been reported previously. TAC for ∼14 days increased afterload and induced cardiac hypertrophy in rats and the extent of hypertrophy was similar in pregnant and non-pregnant animals. Pregnancy influenced a wide range of genes related to heart remodeling and the shift in MHC-expression towards the β- isoform following TAC was more significant in pregnant animals. Pregnancy led to myocyte thickening independent of TAC and the increase in cardiac stroke work after TAC was more pronounced in pregnant animals compared to non-pregnant.

It is widely believed that pregnancy causes physiological cardiac hypertrophy characterized by chamber enlargement without any increase in LV wall thickness/chamber diameter ratio [10], [15], [16]. Pathologically increased afterload in pregnancy, such as in preeclampsia, induces a concentric hypertrophy characterized by increased LV wall thickness to cavity ratio [10].

However, in the present study in rats, pregnancy *per se* did not lead to significant cardiac hypertrophy. Which parameter best describes cardiac hypertrophy in pregnancy remains controversial. Conventionally used parameters at the organ level include HW, LV weight and HW/BW-ratio. As maternal BW increases with advancing gestation, use of HW/tibia ratio [17] may be better than HW/BW ratio. However, none of the parameters measured supported the evidence of significant cardiac hypertrophy in pregnant Wistar rats. These findings are in contrast to some other studies performed in rats [2], [18]–[20], but in line with our previous study in Wistar rats [8]. Our present study had 80% statistical power to detect an increase in HW of 13% due to pregnancy. When all pregnant (n = 24) and non-pregnant (n = 18) sham animals from the present study and our previous study [8] were pooled together there was still no difference in HW between groups (730±20 vs 700±14 mg, p = 0.24). We are not aware of other studies in rats with equally high number of pregnant animals included.

In our study LV internal diameter in diastole (Table 1) and LV end-diastolic pressure (Fig. 4) were not increased in pregnant sham rats, and diastolic BP was *lower* in pregnant rats compared to non-pregnant (Fig. 2). This may explain why the heart size was not affected by pregnancy. Slangen et al have shown increased CO in pregnant Wistar rats using electromagnetic flow probes around ascending aorta [21]. Studies in mice indicate that there is a good correlation in measurements obtain by flow probes and echocardiography [22]. However, in our study M-mode echocardiography did not detect differences in CO between groups. Anesthesia may have affected CO in our experiments, but HW could not have been affected by anesthesia.

As expected TAC increased afterload, but the increase in afterload attributed to TAC was not affected by pregnancy. TAC increased systolic BP but the diastolic BP was not affected. This was in contrast to chronic pressure load caused by AngII where both systolic and diastolic BP increase [8]. The increase in afterload caused by TAC was compensated by an increase in stroke work and this increase was more pronounced in the pregnant animals. This may be related to the fact that pregnancy in combination with TAC led to an increase in myocyte circumference. Additionally, cardiac remodeling following TAC was associated with a more than three-fold increase in the ratio of β-MHC/α-MHC-gene expression in pregnant compared to non-pregnant rats.

TAC is used as a model of chronic pressure overload and non-pregnant rats generally develop progressive concentric LV hypertrophy and ultimately LV dilatation and heart failure [23]. In the present study there were no differences in filling pressure, heart rate, SV, CO and weight gain between TAC and sham operated animals irrespective of whether they were pregnant or not. This indicates that moderate constriction of the transverse aorta for ∼2 weeks is well compensated with LV hypertrophy and increased stroke work (Table 1, Figures 2 and 4). A longer time following TAC could lead to overt heart failure [23], but the short duration of pregnancy in rats (about 21–22 days) did not allow this. Mating non-pregnant animals after TAC or even banding the ascending aorta on juvenile rats [24] could be an option and may be more relevant to simulate a GUCH-situation. This should be considered for future studies. However, the effect of these interventions on fertility is not known.

Women with hypertensive disorders of pregnancy are known to be susceptible to pulmonary edema [25]. A more pronounced increase in cardiac stroke work after TAC in observed in pregnant rats may represent a hypercontractile state with increased myocardial metabolic demand before decompensation leading to overt heart failure [26] and thus making the heart vulnerable to additional stress in pregnancy.

Increased myocyte size in pregnancy has been attributed mainly to increased myocyte length [9], [19] whereas the remodeling due to pressure overload is associated with a greater increase in cardiac myocyte width than length [27]. Our study shows that the circumference of myocytes also increases in pregnancy, but TAC for ∼2 weeks did not increase the myocyte circumference significantly. An increased β-MHC expression and decrease in α-MHC expression has been considered as hallmarks of cardiac hypertrophy [28], [29]. As expected, expression of β-MHC was increased and expression of α-MHC was decreased following TAC. Interestingly this shift in MHC-expression towards the β- isoform was more pronounced in pregnant rats. A recent study in mice has shown that β-MHC protein is induced by pressure overload only in a minor subpopulation of myocytes that are smaller than myocytes containing α-MHC only [30]. This may explain why TAC for ∼2 weeks did not lead to an increase in average myocyte circumference even if the ratio of β-MHC to α-MHC expression was increased.

Previously we have shown that pregnancy inhibits the fibrogenic effect of AngII in rats [8]. In contrast to AngII infusion for ∼10 days, TAC for ∼14 days did not increase relative collagen content in cardiac tissue (Fig. 3), and the increase in the cardiac expression of fibrosis related genes was less pronounced following TAC (Table 2) compared to AngII infusion [8]. This is not surprising because AngII is known to have a direct fibrogenic effect on the heart independent of increase in BP [31]. TGF-β acts as a regulator of cardiac remodeling through its direct actions on the cardiomyocyte, the fibroblast and the extracellular matrix, and is induced by hemodynamic overload [32]. TGF-β1 acts downstream to AngII in inducing myocardial fibrosis [32]. The expression of TGF-β1 and TGF-β3 was reduced in pregnancy (Table 3) which may explain why pregnancy counteracts the development of fibrotic remodeling of the LV in AngII infused rats [8].

Increased expression of ANP and BNP is associated with heart failure. While ANP expression in the myocardium is not affected by pregnancy [2], [8], [9], Jankowski *et al.* found a decrease in BNP expression at term [2]. However, neither the present study nor our previous study [8] could confirm this finding. As in AngII infused rats [8] the increase in ANP and BNP expression after TAC was not affected by pregnancy.

Expression of TNF-α and nitric oxide synthases iNOS and eNOS were reduced by pregnancy in TAC (Table 2). Expression of TNF-α and iNOS are also related to inflammation. Whether the reduction of their expression is associated with reduced inflammatory response of pregnancy [33], [34] or changes in sex hormones during pregnancy [1], [2], [35] remains to be elucidated.

In a study comparing physiological cardiac remodeling in pregnancy with pathological, Eghbali et al. used TAC male mice as controls [9]. In the present study, we used rats of same sex, similar age and size to study the effects of pregnancy and/or TAC on cardiac remodeling. This minimizes confounding and enables to determine if changes in structure, function or gene expression can be attributed to pregnancy, TAC, or both. The study is further strengthened by using two different methods to measure cardiac function (echocardiography and conductance catheter) in the same animal. We used echocardiographic LV volumes instead of the voltage signal from the conductance catheter. By this we avoided injection of a large intravenous bolus of hypertonic saline that is necessary to obtain true volumes by the conductance catheter method.

There are some limitations of our study. *In vivo* measurements of heart function are invariably affected by extrinsic factors. We have performed all our experiments under standardized and controlled conditions. However, differences in the animals' response to anesthesia, degree of volume loading, and temperature are important confounders of particular relevance in pregnancy. However, the heart rate, temperature, end diastolic volume and end diastolic pressure did not differ significantly between groups.

In order to be able to examine both morphology and gene expressions from the same animal, mRNA expression studies were performed on LV apical samples whereas morphological studies were done in mid-ventricular tissue. Ideally samples from the same area of the LV should have been studied as the myocardium from different regions may have differing properties [36].

Even if performed as a standardized procedure, the degree of TAC will vary, and ideally the pressure gradient over the constriction should be measured in all animals. We catheterized the *left* carotid artery in three animals, and in these rats the pressure drop over the aortic constriction was 43% (±16%) in systole and 33% (±17%) in diastole. However, when both carotid arteries were obstructed BP and heart rate fell rapidly, probably as a result of anoxia of the central nervous system. Inserting a catheter in a peripheral artery could have solved this problem, but we chose not to catheterize the femoral artery to reduce the experimental time and risk of destabilization of the animal. After surgery we were not able to obtain reliable flow signals from the aortic arch, probably due to extensive scaring of the connective tissue. Thus echocardiography could not be used to estimate the pressure gradient across the constriction.

HW was significantly increased by TAC. However, neither collagen content nor myocyte transverse circumference was increased. Some of the dissociation between HW and myocyte transverse circumference and collagen content could also be attributed to regional differences in the myocardium as only mid-ventricular tissue was studied. More importantly, cardiomyocyte volume is dependent on both circumference and length. However the myocyte length was not measured as it cannot be reliably measured in tissue sections [37]. As there was no significant increase in extracellular collagen deposition or cardiomyocyte circumference, an increase in cardiomyocyte length is the most probable cause of the increase in heart weight after TAC. None of the functional measurements obtained with M-mode echocardiography revealed significant differences between any groups. Differences in LV relative wall thickness and calculated LV mass corresponded to actual measurements of HW and LV weight, but with less significance. This illustrates the limitations of M-mode echocardiography [13], [38], and these measurements should not replace necropsy measurements when available.

## Conclusions

The present study demonstrates that pregnancy significantly influences cardiac remodeling in response to increased afterload, but does not lead to progression of afterload induced cardiac hypertrophy. Some differences in cardiac structure, function and gene expression observed between pregnant and non-pregnant rats following TAC indicated that afterload increase is less tolerated in pregnancy. This study did not support the hypothesis that pregnancy is cardioprotective against the negative effects of increased afterload.

Further studies are needed to explore the mechanisms of cardiac remodeling in normal and complicated pregnancies that may lead to new therapeutic approaches when dealing with hypertension and cardiac disease in pregnancy.

## Supporting Information

Table S1 **Primers used for real-time polymerase chain reaction analysis.**
(DOC)
