# Supplementary material for: Effect of Transverse Aortic Constriction on Cardiac Structure, Function and Gene Expression in Pregnant Rats
Source: PLoS One. 2014 Feb 20;9(2):e89559. doi: 10.1371/journal.pone.0089559 (PMC3930736; doi:10.1371/journal.pone.0089559)
Supplement: Table S1 — Primers used for real-time polymerase chain reaction analysis. (DOC) [file pone.0089559.s001.doc]

Table S1 Primers used for real-time polymerase chain reaction analysis.

| RAT mRNA | Primer sequence (5’-3’) |
| --- | --- |
| **Housekeeping genes** | |
| ***GADPH: Glyceraldehyde-3phosphate dehydrogenase*** | |
| Forward primer | CTG-CAC-CAC-CAA-CTG-CTT-AC |
| Reverse primer | CAG-AGG-TGC-CAT-CCA-GAG-TT |
| ***B2M: β-2 microglobulin*** | |
| Forward primer | TGC-CAT-TCA-GAA-AAC-TCC-CC |
| Reverse primer | GAG-GAA-GTT-GGG-CTT-CCC-ATT |
| ***Cyclo: Cyclophilin*** | |
| Forward primer | CTG-ATG-GCG-AGC-CCT-TG |
| Reverse primer | TCT-GCT-GTC-TTT-GGA-ACT-TTG-TC |
| ***HPRT: Hypoxanthine phosphoribosyltransferase*** | |
| Forward primer | GAC-CGG-TTC-TGT-CAT-GTC-G |
| Reverse primer | ACC-TGG-TTC-ATC-ATC-ACT-AAT-CAC |
| ***LDHA: Lactate dehydrogenase*** | |
| Forward primer | GAT-CTC-GCG-CAC-GCT-ACT |
| Reverse primer | CAC-AAT-CAG-CTG-GTC-CTT-GAG |
| **Heart function related genes** | |
| ***α-MHC: α-Myosin heavy chain*** | |
| Forward primer | CAA-GGC-AAA-CCT-GGA-GAA-AG |
| Reverse primer | GGG-TAT-AGG-AGA-GCT-TGC-CC |
| ***β-MHC: β- Myosin heavy chain*** | |
| Forward primer | GAG-GAG-AGG-GCG-GAC-ATT |
| Reverse primer | ACT-CTT-CAT-TCA-GGC-CCT-TG |
| ***ANP:*** ***Atrial natriuretic peptide*** | |
| Forward primer | CAA-CAC-AGA-TCT-GAT-GGA-TTT-CA |
| Reverse primer | CGC-TTC-ATC-GGT-CTG-CTC |
| ***BNP:*** ***B-type natriuretic peptide*** | |
| Forward primer | GTC-AGT-CGC-TTG-GGC-TGT |
| Reverse primer | CAG-AGC-TGG-GGA-AAG-AAG-AG |
| ***PKC-α: Protein kinase C-α*** | |
| Forward primer | CAA-GCA-GTG-CGT-GAT-CAA-TGT |
| Reverse primer | GGT-GAC-GTG-CAG-CTT-TTC-ATC |
| ***ANKRD1: Ankyrin repeat domain-containing protein 1*** | |
| Forward primer | GCTGGAGCCCAGATTGAA |
| Reverse primer | CTCCACGACATGCCCAGT |
| ***TNF-α:*** ***Tumor necrosis factor-α*** | |
| Forward primer | GCC-CAG-ACC-CTC-ACA-CTC |
| Reverse primer | CCA-CTC-CAG-CTG-CTC-CTC-T |
| ***TGF-β1: Transforming growth factor β1*** | |
| Forward primer | AAG-AAG-TCA-CCC-GCG-TGC-TA |
| Reverse primer | TGT-GTG-ATG-TCT-TTG-GTT-TTG-TCA |
| ***TGF-β2: Transforming growth factor β2*** | |
| Forward primer | ATC-GAT-GGC-ACC-TCC-ACA-TAT-G |
| Reverse primer | GCG-AAG-GCA-GCA-ATT-ATC-CTG |
| ***TGF-β2: Transforming growth factor β2*** | |
| Forward primer | CCC-GAT-GGC-GAA-AGG-CCG-AG |
| Reverse primer | TAG-GGT-AGC-CGG-AGG-CCC-CT |
| **Angiogenesis related genes** | |
| ***VEGF-α: Vascular endothelial growth factor-α*** | |
| Forward primer | CAA-GCC-AAG-GCG-GTG-AGC-CA |
| Reverse primer | TCT-GCC-GGA-GTC-TCG-CCC-TC |
| ***VEGF-β: Vascular endothelial growth factor-β*** | |
| Forward primer | ACC-AGA-AGA-AAG-TGG-TGT-CAT-G |
| Reverse primer | TGA-GGA-TCT-GCA-TTC-GGA-CTT-G |
| **Oxidative stress related genes** | |
| ***SOD1: Superoxide dismutase 1*** | |
| Forward primer | TTC-GTT-TCC-TGC-GGC-GGC-TT |
| Reverse primer | TTC-AGC-ACG-CAC-ACG-GCC-TT |
| ***eNOS: Endothelial nitric oxide synthase*** | |
| Forward primer | TGA-CCC-TCA-CCG-ATA-CAA-CA |
| Reverse primer | CGG-GTG-TCT-AGA-TCC-ATG-C |
| ***iNOS: Inducible nitric oxide synthase*** | |
| Forward primer | ACCATGGAGCATCCCAAGTA |
| Reverse primer | CAGCGCATACCACTTCAGC |
| **Fibrosis related genes** | |
| ***COL1A1: Collagen type I-α1*** | |
| Forward primer | CAT-GTT-CAG-CTT-TGT-GGA-CCT |
| Reverse primer | GCA-GCT-GAC-TTC-AGG-GAT-GT |
| ***COL3A1: Collagen type III-α1*** | |
| Forward primer | TCC-CCT-GGA-ATC-TGT-GAA-TC |
| Reverse primer | TGA-GTC-GAA-TTG-GGG-AGA-AT |
| ***FN1: Fibronectin 1*** | |
| Forward primer | CAG-CCC-CTG-ATT-GGA-GTC |
| Reverse primer | TGG-GTG-ACA-CCT-GAG-TGA-AC |
| ***TIMP1: Tissue inhibitor of metallopeptidase 1*** | |
| Forward primer | CAG-CAA-AAG-GCC-TTC-GTA-AA |
| Reverse primer | TGG-CTG-AAC-AGG-GAA-ACA-CT |
